# Supplementary material for: Regulation of Inflammation-Mediated Endothelial to Mesenchymal Transition with Echinochrome a for Improving Myocardial Dysfunction
Source: Mar Drugs. 2022 Nov 30;20(12):756. doi: 10.3390/md20120756 (PMC9781361; doi:10.3390/md20120756)
Supplement: Supplementary file 1 [file marinedrugs-20-00756-s001.zip › marinedrugs-2046219-supplementary.pdf]

Supplementary Table S1. Primer sequences used for qRT-PCR

Primer name	Primer Sequence 5'→3'	Tm(℃)
COXII	Forward: GCTGTCCCCACATTAGGCTT<br>Reverse: CGATGGGCATGAAACTGTGG	60
TUFM	Forward: CAGGTACACTAGAGCGTGGC<br>Reverse: TGGAACATCTCAATGCCTGTCA	60
SSBP1	Forward: ATGTGAAAAAGGGGTCTCGAA<br>Reverse: TCCTTCTCTTTCGTCTGGTCA	59
TFB2M	Forward: TGCTATGTCTTCTCGAGGGC<br>Reverse: ATGCGAGTTTCCAAAGTGCC	59
POLG	Forward: GTCGATATTGACCGGTGCCT<br>Reverse: CCAAGGAGCCTTTGGTGAGT	61
TFAM	Forward: TGATTCACCGCAGGAAAAGC<br>Reverse: ACGAGTTTCGTCCTCTTTAGCA	59
D-loop	Forward: CCCTAACACCAGCCTAACCAG<br>Reverse: GGCGGGGGTTGTATTGATGA	61
β-actin	Forward: AGGCTCTTTTCCAGCCTTCC<br>Reverse: CAGGTCTTTGCGGATGTCCA	61
